# A new approach for Y-TZP surface treatment: evaluations of roughness and bond strength to resin cemen

**DOI:** 10.1590/1678-7757-2018-0449

**Published:** 2019-04-11

**Authors:** Marlyni Aparecida ZENS, Alfredo Llerena ICOCHEA, Bruna Carolina COSTA, Paulo Noronha LISBOA-FILHO, Natália Almeida BASTOS, Paulo Afonso Silveira FRANCISCONI, Adilson Yoshio FURUSE, Cesar FOSCHINI, Vicente GERLIN-NETO, Ana Flávia Sanches BORGES

**Affiliations:** 1 Universidade de São Paulo, Faculdade de Odontologia de Bauru, Departamento de Dentística, Endodontia e Materiais Dentários, Bauru, São Paulo, Brasil.; 2 Universidade Estadual Paulista (UNESP), Departamento de Física, Bauru, São Paulo, Brasil.; 3 Universidade Estadual Paulista (UNESP), Departamento de Engenharia Mecânica, Bauru, São Paulo, Brasil.

**Keywords:** Ceramics, Shear strength, Dental air abrasion, Confocal microscopy, Aluminum oxide

## Abstract

**Objective:**

This study aims to evaluate the effect of sonochemical treatment on the surface of yttria-stabilized tetragonal zirconia (Y-TZP) before and after the final sintering.

**Material and Methods:**

Twenty-eight Y-TZP discs were divided into four groups (n=7), according to surface treatment: PRE: pre-sintering sonication with 30% nominal power for 15 min; POS: post-sintering sonication with 30% nominal power for 15 min; JAT: air abrasion with 50-μm alumina particles; and CON: control group with no treatment. The POS and JAT groups were sintered before sonication and the PRE group after sonication. Surface roughness was analyzed using confocal microscopy, after which resin cement cylinders were placed on the surface of the Y-TZP discs and subjected to mechanical microshear bond strength test until fracture. Surface roughness and microshear bond strength values underwent ANOVA and the Tukey tests.

**Results:**

The surface roughness values for the PRE group (299.91 nm) and the POS group (291.23 nm) were not significantly different (p≥0.05), statistically, and the surface roughness value of the JAT group (925.21 nm) was higher than those of PRE and POS (p=0.007) groups. The mechanical microshear bond strength test showed that there was no statistically significant difference between the groups (p=0.08).

**Conclusions:**

Therefore, the results showed that sonochemical treatment modifies the Y-TZP surface and is similar to the well-established sandblasting surface treatment regarding the strength of the bond with the resin cement.

## Introduction

The popularity and aesthetic requirements of full-ceramic restorations are increasing due to their metal-free nature and improved aesthetics. However, their use in long-term fixed partial dentures has been limited.^1^ Full-ceramic restorations evolved with the appearance of high-strength ceramics, which have better mechanical properties and can be used in metal-free restorations in areas with higher occlusal load.^2^ Yttria-stabilized tetragonal zirconia (Y-TZP) has been used in full-ceramic restorations and is considered a high-strength ceramic.^3^ These Y-TZP ceramic restorations have high flexural strength and are widely used in fixed partial dentures.^4^ However, the success of ceramic restorations depends on, among other factors, high retention and appropriate marginal adaptation after luting.^5^

Y-TZP does not contain silica and is resistant to acid etching, so its bond strength with resin cements can be reduced.^6^ Therefore, methods are necessary to improve the bond strength of Y-TZP with resin cement and, consequently, the long-term prognosis of the prosthetic. Some methods include different mechanical and chemical Y-TZP surface treatments.^7^ Some studies have suggested that sandblasting with aluminum oxide particles obtains the best long-term results.^8^ ^-^ ^10^ Sandblasting increases the roughness of the Y-TZP surface and improves the mechanical retention of resin cement.^7^ Nevertheless, this abrasion reduces the flexural strength of zirconia, because microcracks are formed on the ceramic surface^11^, which may promote an earlier phase transformation from tetragonal to monoclinic on the Y-TZP surface.^12^ ^,^ ^13^ A nano-modified surface can resolve this problem. Such a surface may be obtained by sonochemical treatment, whereby the use of sound waves in the Y-TZP surface results in acoustic cavitation produced by the implosive collapse of bubbles,^14^ which potentially modifies the treated ceramic surface. This treatment can improve the adhesion of Y-TZP with resin cement and causes less damage to the Y-TZP surface.

This study aimed to evaluate the effectiveness of sonochemical treatment on the surface of Y-TZP before and after the final sintering. The null hypothesis test was that there would not be a difference in the strength of the bond of Y-TZP with resin cement and the surface roughness after sonochemical treatment.

## Materials and methods

### Sample preparation

**Figure 1:**
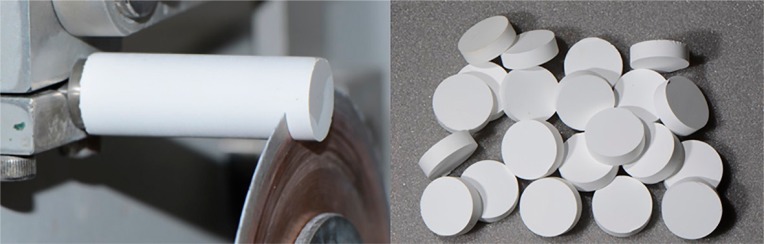
Preparation of specimens by turning and cutting pre-sintered ceramic blocks

**Figure 2:**
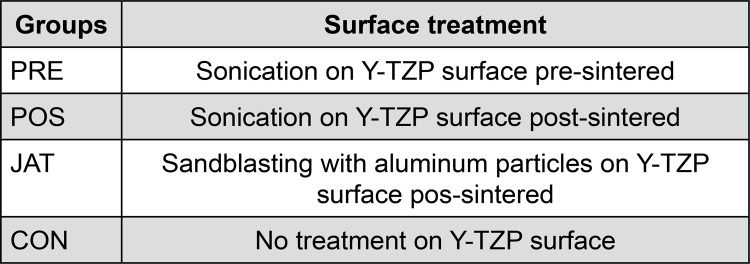
Description of groups according to surface treatment

Twenty-eight discs were obtained from pre-sintering Y-TZP blocks (15.5 mm wide × 19 mm long × 39 mm high) (IPS e.max ZirCAD, Ivoclar Vivadent, Schaan, Liechtenstein), which were milled from a cylinder of 12.5 mm diameter and 39 mm high (Figure 1). Each block was cut using an Isomet 1000 cutter (Buehler, Lake Bluff, Illinois, USA), and a diamond disc (series 15LC Diamond No. 11-4254, Buehler, Lake Bluff, Illinois, USA) was used at 275 rpm under cooling water to obtain the 28 pre-sintering Y-TZP discs (12.5 mm ø and ٣.5 mm thick before sintering) (Figure 1). The discs were randomly divided into four groups according to surface treatment (Figure 2). They were polished in a polishing machine (EXACT, Norderstedt, Schleswig-Holstein, Germany) with #1000 and #1200 sandpapers (Polishing paper K2000, EXACT, Norderstedt, Schleswig-Holstein, Germany), followed by a sequence of treatments on felt wheels with medium, fine, and extra-fine granulations and diamond paste (Polishing paper K2000).

### Surface treatment

**Figure 3:**
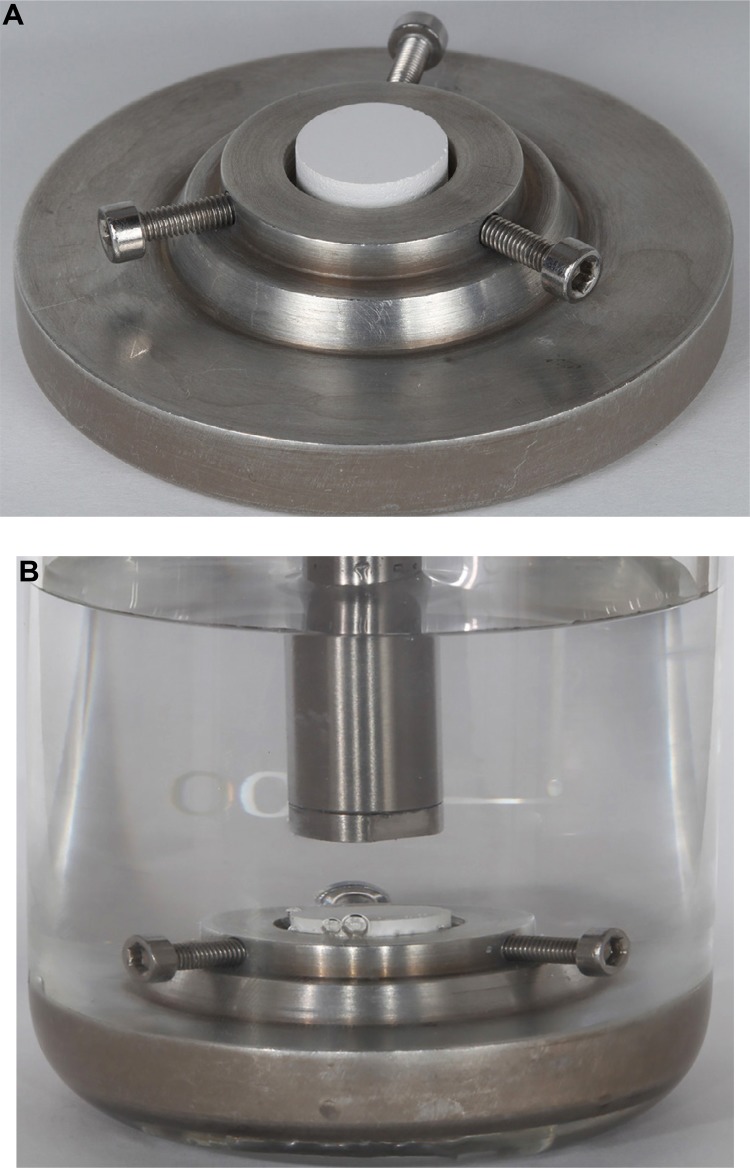
(A) Device used for centering the specimens. (B) Sample in deionized water for treatment with microtip. Centering of Y-TZP discs at the bottom of a beaker containing deionized water

For the PRE and POS groups, the Y-TZP discs were fixed in an ultrasonic processor (Sonics Vibracell VCX-750, Sonics & Materials Inc., Newtown, Connecticut, USA) to standardize their centered position at the bottom of a beaker filled with deionized water, sonochemically treated for 15 min on 30% nominal power (Figure 3). An airborne-particle abrasion device (Microjato, BIO-ART, São Carlos, São Paulo, Brazil) was used to sandblast the JAT specimens with 50-µm-diameter alumina particles under 0.4-MPa pressure perpendicular to and 15 mm from the surface of the disc for 10 s. The discs were cleaned by soaking them twice in 100% ethanol and distilled water in an ultrasound machine (USC 700, Unique Industry and Trade of Electronic Products Ltda, São Paulo, São Paulo, Brazil) for 10 min. The surfaces of the CON group discs were not treated. The POS and JAT group discs were treated after final sintering, while the PRE group discs were treated before final sintering (Figure 2). After the specific surface treatment had been applied, each specimen was sintered in an inFire HTC Speed sintering furnace according to the manufacturer’s instructions (Sirona Dentsply, York, Pennsylvania, USA) at 1530°C for 7 h 52 min. After sintering, the discs dimensions were 10 mm ø and ٢.٨ mm thick.

### Morphological characterization

To evaluate surface roughness and surface topography, the discs in each group (n=7) were analyzed at five sites (scanning area = 400 µm) using confocal microscopy (DCM 3D Model, Leica Microsystems, Wetzlar, Hessen Germany) and an average for each group was calculated.

### Resin cement application

After sintering, the specimens were embedded in a poly(vinyl chloride) cylinder (21 mm in diameter and 25 mm high) using acrylic resin (JET; Classic, São Paulo, São Paulo, Brazil). They were then washed thoroughly with deionized water and dried. A single layer of Single Universal Bond (3M ESPE, St. Paul, Minnesota, USA) was applied to all specimens for 20 s. Then, the specimens were sprayed with oil-free air for 5 s and light-cured for 20 s using a 1100-mW/cm^2^ LED curing light (VALO^®^ Cordless, Ultradent Products, South Jordan, Utah, USA).

**Figure 4:**
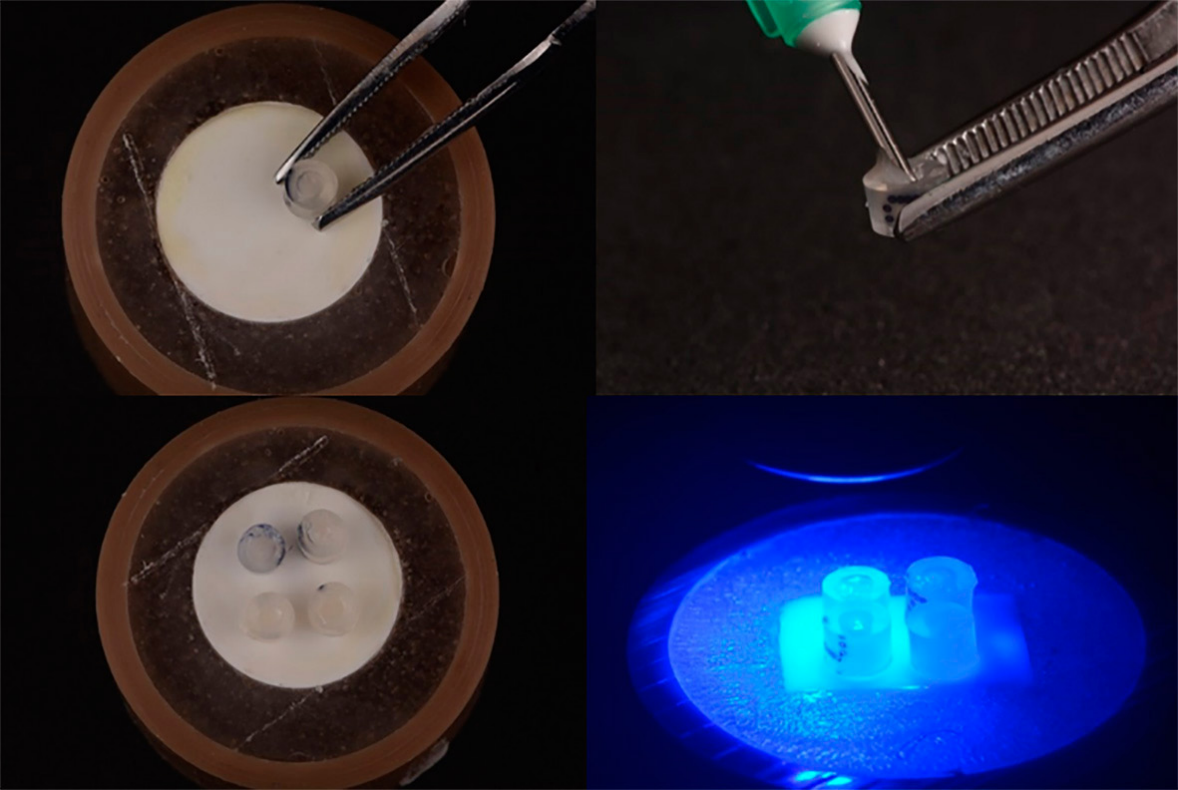
Filling and placement of the catheter tubes with resin cement followed by photoactivation

Adhesion procedures were performed under room temperature and humidity control conditions given in ISO TS11405/2015.^15^ Four surgical catheters (1.40 mm in diameter and 1 mm high) were placed on the surface of each disc to make resin cement tubes (*n*=28). RelyX™ Ultimate Adhesive Resin Cement (3M ESPE, St. Paul, Minnesota, USA), manipulated according to the manufacturer’s recommendations, was inserted into the catheters and polymerized for 20 s with the 1100-mW/cm^2^ LED curing light (Figure 4). After 10 min, the catheters were removed using a No. 11 scalpel blade (Embramed, Jurubatuba, São Paulo, Brazil) to expose the cement cylinders. All cylinders were analyzed with a magnifying glass to verify the absence of defects before undergoing the microshear bond strength (MSBS) test. The specimens were then immersed in deionized water for 24 h at 37°C.^16^

### MSBS test

All 28 specimens were subjected to shear mechanical testing using a universal test machine (Instron, São Paulo, São Paulo, Brazil), with a load cell of 50 kgf. The resin cement cylinders remained aligned with the load cell during testing. A 0.2-mm-diameter steel wire (Morelli Ortodontia, Sorocaba, São Paulo, Brazil) was wrapped around the extension of the load cell of the testing machine and the resin cement cylinder simultaneously. The wire remained in close contact with the lower semicircle of the cylinders and with the ceramic surface. Shear force was applied at a speed of 0.5 mm/min until fracture.

### Statistical analysis

Data on the MSBS test and surface roughness were calculated and statistically analyzed using Statistica software (Statsoft, Tulsa, Oklahoma, USA). The assumptions of normal distribution and equality of variances were checked for all variables using the KolmogorovSmirnov and Levene’s tests, respectively. Because the assumptions were satisfied, the data were subjected to the one-way ANOVA (α=0.05), followed by Tukey’s test (α=0.05) for individual comparisons.

## Results

### Surface roughness

**Figure 5:**
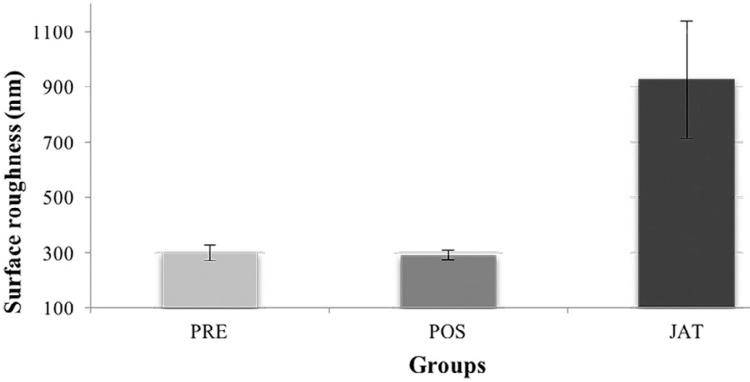
Effect of surface treatment on roughness of Y-TZP ceramic surfaces

There was no statistically significant difference in the confocal analysis of the PRE and POS groups regarding surface roughness. The mean roughness values were 299.91 nm (PRE), 291.23 nm (POS), and 925.21 nm (JAT) (Table 1). The higher superficial roughness values for the JAT group were significantly different (*p*=0.007) than those of PRE and POS, as shown in Figure 5.

**Table 1:** Mean and standard deviation of the roughness results of the groups. Groups identified with same letter are not statistically different (p>0.05)

Groups	Means (nm)	Standard deviation
PRE	299.91^a^	27.38
POS	291.23^a^	17.11
JAT	925.21^b^	213.31

**Figure 6:**
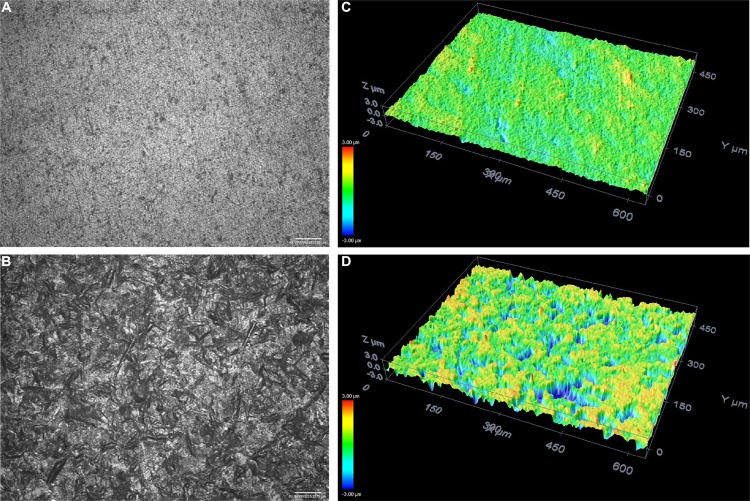
Surface topography shown in 3D images of JAT group before (A, C) and after (B, D) sandblasting treatment (20× magnification). Red represents a vertical (z-axis) size of around 400 nm and dark blue represents 400 nm

**Figure 7:**
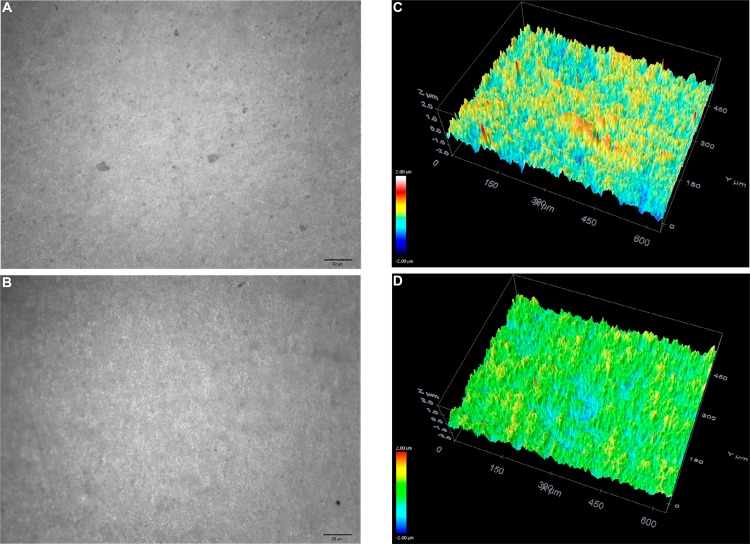
Surface topography shown in 3D images of PRE group before (A, C) and after (B, D) sonication treatment (20× magnification). Red represents a vertical (z-axis) size of around 400 nm and dark blue represents 400 nm

**Figure 8:**
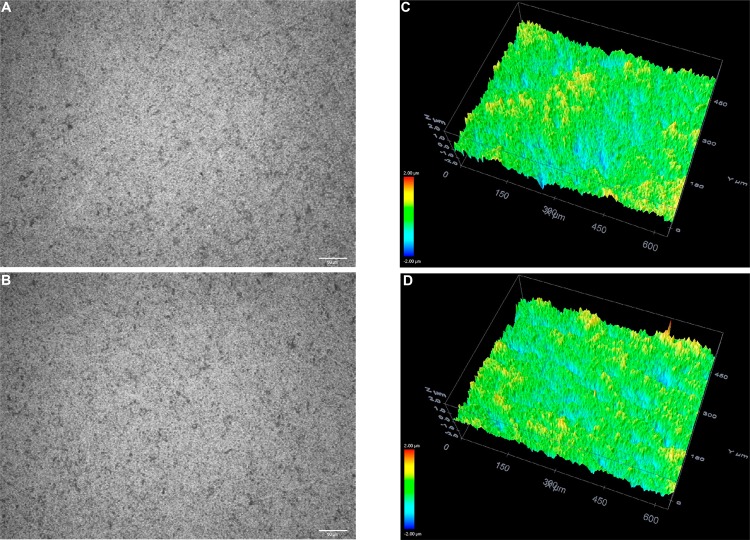
Surface topography shown in 3D images of POS group before (A, C) and after (B, D) sonication treatment (20× magnification). Red represents a vertical (z-axis) size of around 400 nm and dark blue represents 400 nm

Treated surface morphologies were explored in more depth using confocal microscopy analyses. Representative images from these analyses of the zirconia surfaces point to microscale morphological differences (Figure 6). The sandblasted surfaces of the JAT specimens were rougher and more irregular due to the high impact of the alumina particles (Figures 6B and 6D). The sonochemically treated surfaces of PRE (Figures 7B and 7D) and POS (Figures 8B and 8D) groups were more regular compared to the sandblasted surfaces.

### Microshear bond test

**Figure 9:**
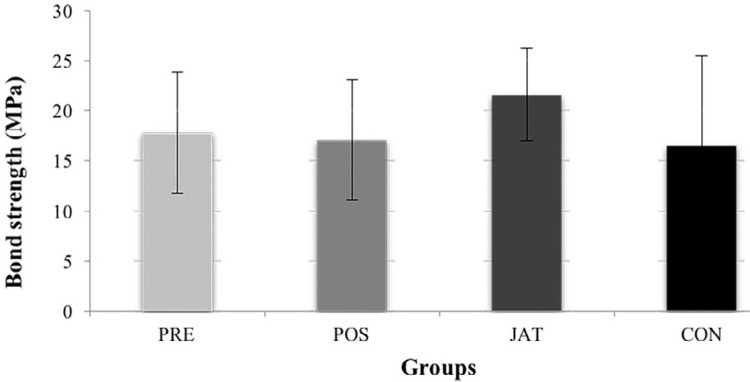
Shear bond strength of veneering ceramics after surface treatments

The initial mean shear bond strength of the CON group (16.48 MPa) was lower than that of the PRE group (17.81 MPa), POS group (17.06 MPa), and JAT group (21.6 MPa) (Figure 9). The Tukey’s test results showed that there was no significant difference (*p*=0.08) in the microshear bond strengths among all groups (Table 2).

**Table 2:** Shear bond strength means (in MPa) for different treatments (n=7). Groups identified with same letter are not statistically different (p>0.05)

Groups	Means (Mpa)	Standard deviation
PRE	17.81^a^	6.06
POS	17.06^a^	6.01
JAT	21.6^a^	4.63
CON	16.48^a^	9.02

## Discussion

We tested the null hypothesis that there would be no difference in the MSBS of Y-TZP to resin cement and in the surface roughness of Y-TZP discs after sonochemical treatment and found that the hypothesis was true for MSBS and false for surface roughness.

Most researchers evaluate different types of surface treatment of Y-TZP ceramic that are already sintered. Although studies have shown that some treatments such as ground + zirconia primer (25.5 MPa), airborne-particle abraded + silanated (22.9 MPa), zirconia primer (22.0 MPa), and airborne-particle abraded + zirconia primer (20.8 MPa)^17^ yield good bond strength of the ceramic with resin cements, these treatments may produce ceramic failures. They can also induce the phase transformation responsible for reducing fracture resistance in both short and long term.^17^ ^-^ ^19^ Therefore, the ideal surface treatment for Y-TZP ceramics should lead to adequate bonding with no risk of damaging the material. Two studies reported an appropriate bond between cement and pre-sintered ceramic without the induction of material phase transformation by the surface treatment.^9^ ^,^ ^10^ Accordingly, we decided to treat the Y-TZP ceramic in two stages in our study: first on pre-sintered and then on sintered ceramic.

The evaluation of the Y-TZP surface roughness without and after surface treatment identified modification by the applied treatments. The increase in surface roughness implies a larger surface area, which is important to increasing the contact between the resin cement and the indirect restoration.^20^ In this study, sonochemical treatment was applied both before (PRE) and after (POS) sintering the ceramic. There was no significant difference between the altered surface roughness of the PRE and POS groups. However, there was a significant difference between the surface roughness of the PRE and POS groups and that of the JAT group.

The lower surface roughness from sonochemical treatment changes the surface on a nanoscale, while sandblasting treatment changes are on a microscale. Both surface changes aim to increase the surface area of the zirconia. However, surface defects from sandblasting can reduce the longevity of a restoration over time^21^ ^,^ ^22^ because of the crystalline phase transformation that occurs on the Y-TZP surface.^12^ ^,^ ^13^

Several laboratory tests, such as shear, tensile, microtensile, and microshear have been used to evaluate bond strength.^23^ The microshear test proved to be the most appropriate. Compared to the shear test, the microshear test decreased the stress on the substrate, thus producing less cohesive failure^24^ and an improved and homogeneous distribution of forces along the adhesive interface.^25^ Likewise, the microshear test allows the evaluation of several specimens simultaneously.^26^ This *in vitro* study has several limitations. Its design makes it difficult to simulate the real conditions of the oral environment and it does not reproduce changes in temperature, loading amplitude, or humidity.^27^

Although the innovative sonochemical treatment did not increase the MSBS between Y-TZP and resin cement compared to other treatments, confocal microscopy showed that it caused less surface damage than sandblasting, suggesting that this treatment would be an effective alternative way to treat the surface of Y-TZP.

## Conclusion

This study has shown that the alternative sonochemical treatment was able to modify both pre-sintering and sintered Y-TZP surfaces. Its findings suggest that sonochemical treatment may be a potential alternative for Y-TZP surface treatment, because the bond strength of Y-TZP to the resin cement after sonochemical treatment was similar to that of the well-established sandblasting surface treatment.
